# Extracellular Vesicles as Delivery Systems in Disease Therapy

**DOI:** 10.3390/ijms242417134

**Published:** 2023-12-05

**Authors:** Manuel Alejandro Picon, Liyong Wang, Andrea Da Fonseca Ferreira, Chunming Dong, George R. Marzouka

**Affiliations:** 1 Interdisciplinary Stem Cell Institute, University of Miami Miller School of Medicine, Miami, FL 33136, USA; map1253@med.miami.edu (M.A.P.); axd1272@med.miami.edu (A.D.F.F.); 2 John T. Macdonald Foundation Department of Human Genetics and the John P. Hussman Institute for Human Genomics, University of Miami Miller School of Medicine, Miami, FL 33136, USA; lwang1@med.miami.edu; 3 Department of Medicine, University of Miami Miller School of Medicine, Miami, FL 33136, USA; 4 Section of Cardiology, Department of Medicine, Miami VA Health System, University of Miami, Miami, FL 33125, USA

**Keywords:** extracellular vesicles, therapy, CRISPR/Cas9, cancer therapy, drug delivery, vaccines

## Abstract

Extracellular vesicles (EVs)/exosomes are nanosized membrane-bound structures that are released by virtually all cells. EVs have attracted great attention in the scientific community since the discovery of their roles in cell-to-cell communication. EVs’ enclosed structure protects bioactive molecules from degradation in the extracellular space and targets specific tissues according to the topography of membrane proteins. Upon absorption by recipient cells, EV cargo can modify the transcription machinery and alter the cellular functions of these cells, playing a role in disease pathogenesis. EVs have been tested as the delivery system for the mRNA COVID-19 vaccine. Recently, different therapeutic strategies have been designed to use EVs as a delivery system for microRNAs and mRNA. In this review, we will focus on the exciting and various platforms related to using EVs as delivery vehicles, mainly in gene editing using CRISPR/Cas9, cancer therapy, drug delivery, and vaccines. We will also touch upon their roles in disease pathogenesis.

## 1. Introduction

EVs/exosomes are naturally produced by almost all cells to facilitate cell–cell communication. EVs have an enclosed lipid bilayer and are known to pack a wide array of bioactive molecules such as peptides, lipids, and nucleic acids that protect them while delivering them to the target cells, transmitting/propagating information [1]. The cargo of the EVs depends on the parental cells and the pathophysiological circumstances where the EVs are released. EV cargo allows the parental cells to exert their functions in neighboring cells/tissues as well as at distant sites. In addition, the topography of the membrane protein on the EV surface allows for EVs to target specific tissues and to facilitate the uptake of the EVs by the recipient cells [2]. Importantly, the biocompatibility and specificity of EVs have been shown to reduce the untoward side effects associated with systemically delivered drugs [3]. Recent studies have shown that EVs can be isolated and manipulated to target desired tissues with the desired cargo to deliver a vast selection of therapeutics. For these reasons, EVs have gained attention as versatile delivery systems that have the potential to improve the efficacy of therapies through more efficient dose administration and precise delivery to the target tissues. The use of liposomes as a delivery vehicle was essential for the success of the SARS-CoV-2 mRNA vaccine, illustrating the importance of these tools in modern medicine. The information discussed in this review regards promising, exciting research on a variety of approaches that employ EVs/liposomes in therapy and immunology, namely on the delivery of clustered regularly interspaced short palindromic repeats (CRISPR) and the CRISPR-associated protein (Cas 9), vaccines, drugs, and cancer therapies. 

## 2. Extracellular Vesicles 

EVs/exosomes can be classified into three main categories based on size: exosomes, microvesicles, and apoptotic bodies [4]. EVs are involved in a variety of biological processes, including intercellular communication, immune modulation, and disease pathogenesis [5]. Their ability to transfer biological molecules, such as proteins, lipids, and nucleic acids, between cells is facilitated by a variety of mechanisms, including endocytosis, receptor-mediated uptake, and fusion with the target cell membrane [6]. EVs protect their cargo from degradation with enzymes, such as nucleases and proteases, making them an effective means of delivering bioactive molecules to target cells [6]. 

There are many efforts to streamline the criteria to define EVs and to explore their vast potential. The International Society for Extracellular Vesicles (ISEV) released a statement outlining the minimal experimental requirements for defining EVs and their functions [7]. The statement emphasized the need for characterizing EVs using multiple techniques, reporting size distribution, morphology, and cargo content, and standardizing isolation protocols. Adhering to these guidelines has accelerated our understanding of EV biology and their potential clinical applications through collaborative efforts from the scientific community. For example, it is important to report the proteins found in isolated EVs to help characterize and determine whether the protein composition matches target expectations [7]. Researchers must also consider the biological context in which EVs are studied. 

EVs are implicated in a range of physiological and pathological processes. For example, cancer cells release EVs that can promote tumor growth and metastasis, while immune cells release EVs that can modulate the immune response [3]. Researchers have been investigating the potential of EVs as diagnostic and therapeutic tools, as they have been shown to carry disease-specific molecules that could be used as biomarkers, or as a means of directly delivering therapeutic agents to affected tissues [7]. Overall, the diverse functions and characteristics of EVs highlight their importance in a wide range of biological processes, and the potential for their use in a variety of clinical applications. It is noteworthy that EVs differ from liposomes in that the latter are artificially synthesized lipid bilayer vesicles and are usually >100 nm in diameter, whereas EVs are naturally generated and released by cells and are much smaller (30–100 nm) [8]. Furthermore, EVs are more biocompatible than liposomes. As such, liposomes are more likely to trigger immune responses [8]. However, liposomes are easier to produce and purify than EVs. 

## 3. Modes of EV Modification to Improve Gene/microRNA/Drug Delivery

**Figure 1:**
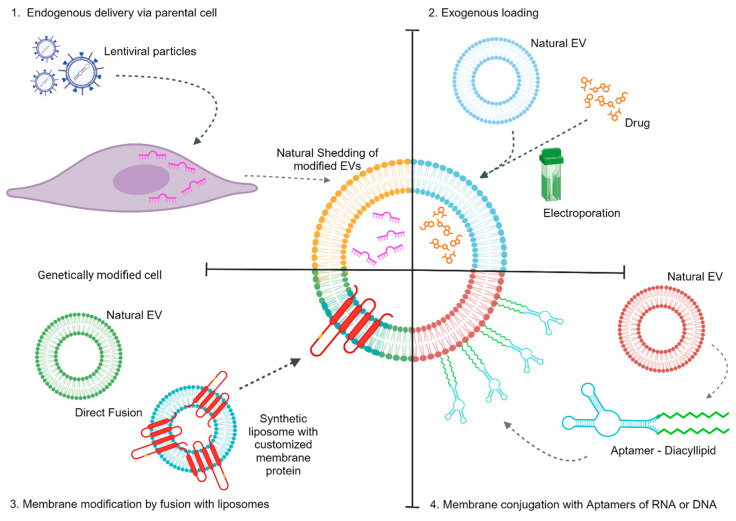
Different examples of strategies used to modify EVs for therapy. Modifications can be performed in EVs’ cargo or membrane with 1. endogenous delivery via parental cells, 2. exogenous loading, 3. membrane modification by fusion with liposomes, and 4. membrane conjugation with aptamers of RNA or DNA.

There are four main types of methods of producing EVs with a modified expression of target genes/miRs and drugs. These include (1) endogenous delivery via parental cell modification, (2) exogenous loading, (3) membrane modification by fusion with liposomes, and (4) the use of aptamers of RNA and DNA. Examples of these different strategies are graphically represented in Figure 1. Endogenous loading is when the source cell of the EV is changed, usually through transfection, to produce desired EVs with modified contents. These modifications can be made to the cargo of EVs or to their membrane for specific tropism. An interesting example is the overexpression of the Rabies virus glycoprotein (RVG), a protein that targets the nicotinic acetylcholine receptor to increase the delivery of encapsulated SiRNA in the brain. RVG was fused to the lysosome-associated membrane protein 2b (Lamp2b) of dendritic cells, which is normally associated with the EV membrane [9]. EVs produced by these modified dendritic cells expressed high levels of Lamp2b fused to the neuron-specific RVG peptide on their surface, allowing the EVs to accumulate in the brain through the interaction between RVG and the nicotinic acetylcholine receptor [9]. 

Exogenous loading is the manipulation of EVs once they have already been isolated from the source cell to modify their cargo [10]. This is usually achieved via electroporation, sonication, freeze/thaw cycles, among others [11]. Typically, hydrophobic drugs do better with exogenous integration while hydrophilic drugs are better incorporated using endogenous loading [11]. EVs can be loaded with many different biochemical molecules [12,13], which allows for versatility in diseases that can be treated through the use of EVs. EVs have also been effective in passing through the blood–brain barrier and treating neurodegenerative disorders, like Parkinson’s and Alzheimer’s disease, with better efficacy. However, optimal drug loading is still a challenge. EVs are also difficult to isolate and produce in sufficient quantities for disease treatment, contributing to the delay of translating EVs to clinical use [4,11]. The ability to avoid degradation in the human body is also important to increase the circulation time of EVs. As described above, CD47 is a membrane surface protein that protects EVs from being phagocytized by macrophages. EVs with higher levels of CD47 have been proven to lengthen their half-lives, allowing EVs more time to deliver their target genes, miRs, and encompassed drugs [10].

In modification studies that relied on fusion with liposomes, hybrid EVs that used polyethylene glycol (PEG) to induce fusion between EVs and liposomes have been developed to improve drug delivery efficiency [14]. It was observed that the fusion of the EVs and liposomes did not result in loss of the drug concentration and that the loading was efficient inside the hybrid EVs, with the highest efficiency at an EV/liposome ratio of 9/1 [14]. These hybrid EVs were highly effective in delivering an anti-tumor drug, mTHCP. Compared with regular EVs, the hybrid EVs had a significantly higher percent of the drug loaded, 3% and 90%, respectively [14]. In addition, the spontaneous fusion of natural EVs and synthetic liposomes with engineered membranes that target specific tissues also generated promising results [15]. Recent advances have focused on the use of aptamers to deliver EVs. In these approaches, DNA [16] or RNA aptamers [17] are used. Aptamers can bind to diacyllipids, which, in turn, naturally bind to the EV membranes. The aptamers are specifically designed to bind proteins in specific cell types, allowing for the targeted delivery of the EVs to these cells. 

## 4. EVs in CRISPR Delivery

Clustered regularly interspaced short palindromic repeats (CRISPR) and the CRISPR-associated protein (Cas 9) have proven to be a revolutionary gene engineering tool with vast therapeutic potential [18]. The gene editing ability of CRISPR/Cas9 has been shown to be effective in upregulating fetal hemoglobin production in patients with Beta-Thalassemia and sickle cell disease [19]. It has been used to form chimeric antigen receptor T cells (CAR-T cells) to treat refractory forms of acute lymphoblastic leukemias [20]. EVs have been investigated as an effective delivery method to deliver the CRISPR/Cas9 system to the desired cells for gene editing [2]. However, two issues concerning the use of EVs for CRISPR/Cas9 delivery have been the delivery efficiency of EVs and the loading efficiency of the CRISPR/Cas9 protein into the EVs.

### 4.1. Maximizing CRISPR Loading in EVs

To circumvent the problem with plasmid expression in the target cells, a different strategy of loading the CRISPR/Cas9 complex consists of loading the expressed CRISPR/Cas9 protein and single-guided RNA (SgRNA) into an EV. To increase the efficiency of loading large-protein cargo, such as the CRISPR/Cas9 complex, into EVs, the Cas9 protein was heterodimerized with a heterodimer partner fused with the EV’s naturally associated proteins, e.g., CD9 and CD81, or a heterodimer partner modified with a fatty acid moiety [21]. The EV-sorting motifs facilitated membrane localization and preferential packing of the heterodimerized complex into EVs. Furthermore, reversible dimerization systems were used to allow active loading during EV production and release of the cargo in the recipient cells. The reversible dimerization process was either controlled by light or a small molecule. Without the EV-sorting motif, there was negligible Cas9 complex loading into the EV. Both CD9 and Myristoylation–Palmitoylation–Palmitoylation lipid modification were successful in loading Cas9 into EVs and in preserving the functionality of Cas9 [21]. In addition to the heterodimer system, another strategy to fuse CRISPR/Cas9 to an EV-sorting motif is to use a short single-stranded signaling ribonucleotide, called an aptamer, and an aptamer-binding protein (ABP) to facilitate an interaction between the two proteins and increase the loading of CRISPR/Cas9 inside the EVs [22]. The RNA aptamer sequence was embedded into the sgRNA, while CD63, an EV marker, was fused with the ABP at both ends of the protein. With this strategy, it is important to express vesicular stomatitis virus G (VSV-G) protein to facilitate the breakdown of the EV to release the CRISPR/Cas9 complex, once it is inside the recipient cell [22]. 

### 4.2. CRISPR Delivery for Cancer Treatment

The delivery of CRISPR/Cas9 via EVs has shown success for treatment against different types of cancer. EVs transfected with CRISPR/Cas9 DNA plasmids were delivered to the desired target cancer cells. Once inside the target cell, the CRISPR/Cas9 complex was translated and assembled [23]. One study showed moderate knockdown of the KRAS gene in pancreatic cancer cells and the reduction of pancreatic tumor size with the use of EV-delivered CRISPR/Cas9 plasmids [23]. Additionally, breakthrough cancer treatments, such as Chimeric Antigen Receptor (CAR) T-Cells, have been combined with EVs to improve CRISPR delivery to B-cell malignancies. CARs are genetically modified receptors that are specific to cancer antigens used to increase the specificity of treatment [24]. One group took advantage of this technology by modifying the surface of CRISPR containing EVs with a CAR specific to B-cell malignancies. In this case, the CAR was made to target CD19; this tumor antigen was used due to its high expression in B-cell malignancies [24]. Also, the CRISPR/Cas9 used was engineered to target the MYC gene, a Proto-Oncogene. This study found that mice injected with the anti-CD19 CAR EVs loaded with MYC-CRISPR had slowed tumor growth compared with those injected with MYC-CRISPR-loaded EVs without CAR modifications, and the tumor cell damage was observed in immunohistochemical studies [24]. 

### 4.3. Genetic Diseases and CRISPR

Currently, research is being done to use EV- or liposome-delivered gene editing molecules to treat diseases that are linked to genetic mutations. Liposomes were used to improve the delivery of Phosphorodiamidate Morpholinoligomers (PMOs) to treat Duchenne Muscular Dystrophy (DMD) [25]. PMO is the first antisense oligonucleotide approved by the FDA to treat DMD. However, skeletal muscle cells are known to be difficult to transfect/transduce [25]. To improve the delivery efficiency, the A2G80 peptide was added to the liposomes surface as A2G80 peptide binds with substantial affinity to α-dystroglycan expressed on muscle cell membranes. When A2G80 modified liposomes were injected into mice with DMD mutation, this strategy showed a significant accumulation of modified liposomes inside muscle cells and increased association with alpha-dystroglycan inside DMD model mice (mdx mice) [25]. Furthermore, when A2G80-modified liposomes were coated with long- and short-chain PEG, called A2G80-LSP-Lip, these liposomes improved the blood circulation of the liposomes using microfluidics. When the liposomes were administered to mdx mice via the tail vein, A2G80-LSP-Lip accumulated efficiently in the muscle tissue compared with control liposomes [25]. In another study, the CRISPR/Cas9 compound was delivered via transferrin-receptor-binding-peptide-conjugated EVs to knockdown P-glycoprotein (P-gp) in the blood–brain barrier (BBB), ultimately reducing drug resistance in the brain. P-gp is an efflux pump expressed by brain endothelial cells that pumps drugs from the brain endothelial membrane and cytosol compartment back into the blood for subsequent elimination, which makes the brain resistant to certain drugs [26]. This study showed that EV-delivered CRISPR/Cas9 was effective in inhibiting P-gp, making it easier for medications that treat diseases of the brain like Alzheimer’s, amyotrophic lateral sclerosis (ALS), epilepsy, among others, to cross the BBB with improved efficiency [26].

## 5. EVs in Vaccine Delivery 

Vaccines have historically been an effective tool in preventing and eradicating deadly infectious diseases worldwide [27,28]. As technology continues to advance, different vaccine strategies are being researched to improve vaccine effectiveness [29]. EVs have been used as carriers to improve the delivery of vaccine antigens and enhance the immunogenic effects with low reactogenicity [30]. In 1974, it was shown that using liposomes as a vaccine adjuvant in making a liposomal diphtheria toxin vaccine generated significant immunogenic effects [30]. Since then, there have been many efforts to investigate the use of liposomes in vaccines for different diseases.

**Figure 2:**
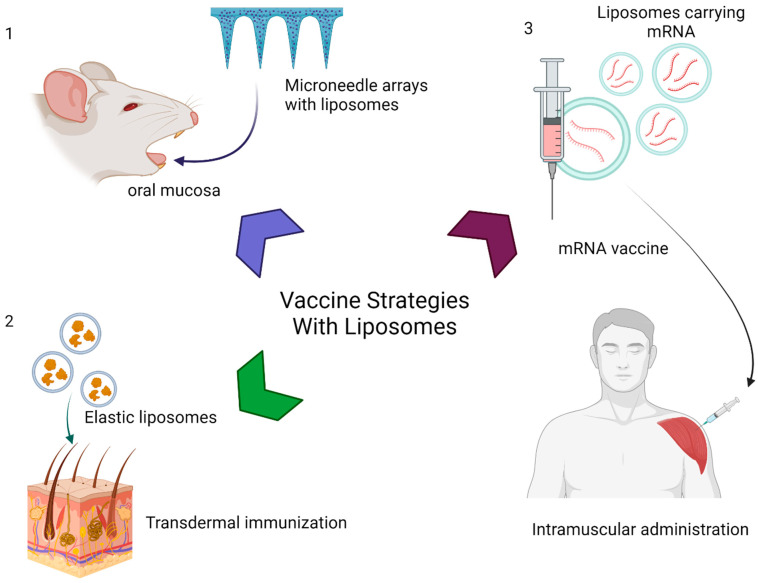
Different strategies employ liposomes in vaccines: 1. microneedle arrays with liposomes for oral mucosa administration, 2. transdermal immunization with elastic liposomes, and 3. mRNA vaccines.

In 2014, an oral hepatitis B vaccine was developed with the intention of creating a painless and needleless effective vaccine [31]. In this strategy, liposomes were incorporated to microneedle arrays as graphically represented in Figure 2(1). Briefly, liposomes were mixed with a polymer in gel form. This material was dried and molded into structures that were implanted in the oral mucosa for liposome delivery. A stable liposome containing the hepatitis B antigens was able to produce a cellular and humoral response when given via the oral mucosal route [31]. Furthermore, an increased level of immunoglobulins and an enhanced Th1/Th2 CD4+ T cell response were observed, primarily due to the liposome containing the C-type lectin-targeting molecule, mannose-PEG-cholesterol conjugate (MPC), and adjuvant lipid A, which are the hepatitis B antigens that induce an immune response [31].

In another study, a transdermal vaccine against *Plasmodium falciparum*, the protozoan parasite associated with malaria, was made with elastic liposomes [32] (represented in Figure 2(2)). These liposomes are prepared by mixing an ethanolic solution of soybean phosphatidylcholine (SPC) with span 80 in PBS (86:14% (*w*/*w*) containing Ag-MSP-1_19_ solution (10 μg/mL). The resulting liposomes were selected for the desired size with a series of filtrations [32]. These liposomes were loaded with merozoite surface protein-1 (PfMSP-1). The study compared different forms of vaccine formulations against PfMSP-1 and the elastic liposome vaccine showed a greater IgG1/IgG2a ratio compared with the other vaccination methods [31]. The study also noted that elastic liposomes increased the levels of IFN-γ compared with those of other vaccines [32]. IFN-γ has been shown to play a crucial role in T-cell immunity and specifically control immune responses against blood-stage malaria [32].

A more recent study examined the efficacy of using liposome vaccines to induce immunity against more common infections like influenza A and streptococcus, using animal models [33]. It was shown that liposomal vaccination against the M2e antigen of influenza A was effective in producing IgA antibodies and decreasing viral titers in the lungs of mice [33]. Similarly, ferrets that received the liposomal vaccine had 90% viral titer reduction compared with those receiving an empty liposome vaccine upon H1N1 infection [33]. This study was also the first to show protection against superinfection using a combined epitope liposomal vaccine [33]. They combined the J8 epitope, an antigen of streptococcus, and the M2e epitope into a single liposome. Vaccination with combined-epitope (multi-vax) liposomes produced IgA levels for each antigen equal to the influenza A liposomal vaccine alone and the streptococcus liposomal vaccine alone. Mice with the multi-vax had much better clinical outcomes when inoculated with influenza A and streptococcus compared with the mice with no vaccination [33]. 

The most relevant recent use of liposomal vaccines has been its utility in the SARS- COVID-19 mRNA vaccine [34,35,36] (Figure 2(3)). The COVID-19 mRNA vaccines elicit immune responses via mRNAs that encode viral antigens and are intramuscularly administered. Tsai et al. used a combination of mRNA-encoding SARS-CoV-2 spike antigens encapsulated in EVs containing LSNME, a fusion of SARS-CoV-2 nucleocapsids, and fragments of spike to inject mice [37]. This combination generated a significant number of antibodies towards spike proteins and nucleocapsids [37]. Another strategy for the making of the SARS-CoV-2 vaccine involved conjugating the spike recombinant receptor binding protein to 1,2-distearoyl-sn-glycero-3-phosphoethanolamine-poly(ethy lene-glycol)-*N*-hydroxysuccinimide, and the conjugated molecule was placed on the surface of EVs. When this EV-based vaccine was given to mice via a nebulizer, it generated high levels of circulating IgG and mucosal IgG and IgA antibodies against SARS-CoV-2 as well as stimulated a robust T cell immune response [38].

## 6. EVs in Cancer Therapy

Cancer therapies are limited due to their lack of selectivity for cancer cells. Current cancer therapies are known to also damage all types of rapidly dividing healthy cells, causing many unwanted side effects [10]. To improve cancer therapy selectivity and achieve targeted delivery, researchers have turned their attention to using EVs as drug carriers. The biocompatibility and natural cell signaling role of EVs in the human body allow them to be efficient drug carriers to cancer cells [10]. In addition, EVs have the ability to target specific organs or tissues using specific surface markers. For example, CD47 protein is highly expressed in many cancer cells. It suppresses the phagocytic function of macrophages and dendritic cells [39]. Antagonizing CD47 via antibodies with nonspecific delivery was associated with serious side effects, inducing anemia and thrombocytopenia, albeit with potent antitumor efficacy. EVs harboring signal regulatory protein alpha (SIPRα)-EV-SIRPα and anti-CD47 with minimal toxic effects on hematologic parameters were developed [40,41]. SIPRα is a naturally occurring regulatory protein usually found on the surface of macrophages. It is known to be a ligand of CD47 that, once bound, causes the inhibition of phagocytosis [42] These EV-SIRPαs used red blood cells as delivery vehicles to tumors, effectively inhibiting ligation of residual CD47 molecules and inducing tumor-specific T-cell-mediated antitumor effects, without inducing apparent anemia [40,41]. 

EVs tend to resemble the properties and signaling surface molecules of the cells they originate from [37]. Tumor cells primed with interferon regulatory factor 1 (IRF-1), a transcription factor associated with antitumor immunity, secrete EVs containing high levels of IRF-1 target genes, MHC I, and IL-15Rα [42]. These EVs stimulated active and specific cellular immunity against tumor cells. Hepatic tumors inoculated with IRF-1-primed-tumor-derived EVs slowed the growth of the tumors [42]. TNF-related apoptosis-inducing ligands (TRAILs) are apoptosis inducing proteins. To increase the bioavailability and efficient delivery of TRAILs, mesenchymal stromal cells (MSCs) were transduced, resulting in the production of TRAIL-containing EVs. Interestingly, this approach also caused an overall increase in EV production by the MSCs [43]. Remarkably, the TRAIL-containing EVs showed superior apoptosis inducing capabilities in cancer cells when compared with the alternative, recombinant TRAIL (rTRAIL) [43]. Liposomes, similar to EVs, have also proven to be successful in delivering cancer therapies. A 2020 study by Pankaj Dwivedi et al. [44] designed unique liposomes containing citric-acid-stabilized magnetic nanoparticles and loaded these liposomes with doxorubicin (DOX), a cancer therapy medication, to form a DOX-loaded magneto liposome (DOX-ML). In addition, they took it one step further and conjugated the DOX-ML to perfluorocarbon (PFC)-loaded microbubbles (MBs). The idea behind this complex liposome was to use an external magnetic field to guide the magneto liposomes to the target tissue and use ultrasound (US) waves to burst the PFC-filled MBs to increase the efficacy and selectivity of DOX administration in pancreatic cancer [44]. They tested different combinations of DOX-ML, US, magnet, and MBs and recorded tumor volume over 20 days and observed that DOX-ML-MB+US+magnet was the only combination to shrink the pancreatic tumors [44]. Upon apoptosis analysis, DOX-ML-MB+US showed the highest number of apoptotic cells compared with the other combinations and the control [44]. This strategy was effective in targeting specific tissues of interest, with increased accumulation of magnetic liposomes in the tissues where the external field was applied when compared with other areas with no electric field [44]. Furthermore, some immune cells were exploited to produce EVs with the inherent immunogenic activity associated with these cells. Specifically, Natural Killer (NK) cells produce EVs containing granzyme B, perforin, and FasL, which are apoptosis-inducing proteins [3]. For example, when NK cells were primed with IL-15, a cytokine that stimulates immune activity, increased production of EVs with elevated concentrations of granzyme B, perforin, and FasL were observed [3]. These EVs showed a significant antitumor activity against glioblastoma, with 89.8% apoptosis of the glioblastoma cells, as compared with the 20.4% apoptosis when the cancer cells were treated with FasL inhibitors [3].

It is noteworthy that EVs with mRNA loading have been used for other disease treatments. For example, EVs loaded with Neprilysin, a membrane-bound metallopeptidase and one of the major amyloid β-degrading enzymes, were effective in decreasing abnormal aggregated beta-amyloid sheets in the brain in a rat model for Alzheimer’s disease [45]. We focused on cancer therapy in this article because of disease prevalence and the intensity of the research in this arena. 

## 7. EVs, microRNAs, and Disease Pathogenesis/Treatment

In contrast to mRNAs, each microRNA (miR) could regulate multiple genes, playing greater roles in disease pathophysiology [46]. There is abundant evidence showing that miRs play important roles in signal transductions, cellular processes, and disease pathogenesis [46,47,48]. We have shown that miRs play important regulatory roles in endothelial progenitor cell senescence and cardiovascular disease (CVD) development [49]. Importantly, miRs and their antagomirs are conceivably easier to deliver to EVs due to their short sequences than mRNAs [50]. Because of these reasons, our group has focused on modification of EVs with miRs. Indeed, we were the first to use genetically modified MSCs to produce tailored EVs (TEVs) loaded with specific miRs to target signaling events underlying disease processes. For example, miR-126 is critical for angiogenesis and CVD development. We have shown that the miR-126-containing TEVs rejuvenated senescent endothelial progenitor cells, increasing their proliferation and differentiation. When injected in aged apoE knockout mice, these TEVs promoted angiogenesis and improved blood supply [51]. In a recent study, we demonstrated that let-7b and miR-103-3a encompassed in EVs isolated from people living with HIV (PLWH) mediated the effects of HIV infection in CVD development and that TEVs loaded with the antagomir for let-7b-5p (miRZip-let-7b and miRZip-103-3a) counteracted the effects of HIV infection on accelerated atherosclerosis [52]. 

## 8. Tissue Distribution of Therapeutic Extracellular Vesicles

Tissue distribution studies have highlighted that EVs tend to accumulate in organs rich in blood vessels and macrophages, such as the liver, lungs, spleen, and kidneys [53]. Multiple factors affect tissue distribution. These include the size of the EVs, the route of the administration, and to a lesser degree, the timing of the treatment, among others. 

The size of the EVs has shown to be a major factor that determines their tissue distribution. The size of EVs are classified as either small or large, small EVs being <100 nm and large EVs being >200 nm. Studies evaluated the concentrations of small EVs at 1 h and then 2–12 h post-intravenous (IV) administration in different organ systems; the concentration eventually declined in a time dependent manner. They found that these particles primarily accumulated in the liver. In the liver, the EV concentration peaked at 1 h and again between 2–12 h, eventually disappearing by hour 12 [54,55]. They also reached peak concentration in the lungs within the first hour but quickly declined after 2–12 h. The spleen showed moderate concentrations of the small EVs at both time intervals, and the kidneys only had low levels of small EVs in the first hour. In contrast to small EVs, large EVs primarily distributed to the lungs 1 h post-IV administration and declined in 2–12 h. Large-EVs also demonstrated gradual accumulation in the liver, peaking at 24 h post-IV administration with low to no levels in the spleen [55].

In addition, the route of administration was shown to affect the tissue distribution of EVs. The common routes that have been tested are intranasal, intravenous, subcutaneous (SC), and intraperitoneal (IP). In SC injections, EVs must encounter adipose tissue, blood vessels, and eventually enter the circulation via lymphatic vessels. However, the distribution is reliant on the ability of the EVs to diffuse through the extracellular matrix (ECM). Furthermore, it is known that the lymphocyte-mediated clearance limits the amount of EVs that can reach the intended destination [56]. IP injection poses issues similar to the SC injections. In IP injections, EVs must cross multiple layers of connective tissue and enter the circulation via the lymphatic system [56]. With regards to local administrations, such as in the intramuscular or intranasal methods, EVs have been shown to remain localized in the tissue in which it is injected, with little spread to systemic circulation [55,56]. This has been attributed to the tight endothelial barriers in these tissues that do not allow the EVs to get into the circulation [55]. Lastly, intravenous administration has been shown to primarily accumulate in the liver and then spread to the lungs, spleen, and kidneys [53,54,55].

Although targeting specific tissues with EVs has been a challenge, some methods of EVs modification have shown promise, as discussed in the previous section. Other authors have proposed the use of EVs in specific contexts that would naturally facilitate their migration to the desired site. In one experiment, Tieu and colleagues induced acute lung injury in mice and administered EVs 24 h after the induction of injury—a timepoint in which inflammation was measured to be at its peak [57]. This study showed a 2.7–4.4-fold increase in EV concentration in the inflamed lungs compared with those in normal non-inflamed lungs [57]. Although this model showed good results that could be the basis for developing therapies for inflamed tissues, other types of pathologies may require more complicated approaches other than the timing of the treatment. These include the manipulation of EVs markers which can allow them to accumulate in desired tissues/organs and be taken up by specific cells to produce specific effects [58]. 

It is important to note that even if advances have been made in the field, targeting strategies seem to be limited to an increase in the concentration of EVs in the desired site of delivery for a certain period of time. This is because EVs persistently disperse to non-target tissues. Perhaps the combination of different strategies of EV targeting would help control tissue distribution and generate better results in the future. 

## 9. Conclusions

In conclusion, EVs have gained significant interest in the field of biomedical research due to their unique properties, including their ability to serve as carriers of therapeutic agents. The use of EVs for the delivery of different therapeutic agents has shown promise to improve the treatment of different diseases. EV delivery of CRISPR/Cas9 has shown great potential in the treatment of various genetic diseases, like Duchenne Muscular Dystrophy [5]. A more recent and ubiquitous example has been the use of EVs’ role in the development of the COVID-19 vaccine [59]. 

EVs have proven to be effective in delivering a multitude of drugs. Specifically, they have been very successful in controlling cancer growth via the improved delivery of cancer therapies [37]. Although challenges remain, such as the efficient loading of therapeutic agents into EVs, the production of EVs in large quantities, and their targeted delivery to specific cells [51], advancements in this area are accelerating. As our understanding of the biology and function of EVs increases, we can expect to see continued progress in the development of novel therapies based on these unique extracellular vehicles.
